# Supplementary material for: Are carbon emissions trading and green financial instruments synergistic? -Comprehensive quantitative research based on content analysis
Source: PLoS One. 2024 Mar 7;19(3):e0298601. doi: 10.1371/journal.pone.0298601 (PMC10919601; doi:10.1371/journal.pone.0298601)
Supplement: S1 Data — (ZIP) [file pone.0298601.s001.zip › Data/地级市Z_F工作报告.zip!/╕╩╦α/╟∞╤⌠╩╨/└┤╫╘╣1⁄2╓┌║┼í╛┬φ┐╦╩2╛▌═°í┐_2019.docx]

各位代表：

现在，我代表市人民政府向大会作工作报告，请予审议，并请各位政协委员和列席人员提出意见。

2018年工作回顾

2018年是改革开放40周年。改革开放40年来，我市经济社会发展突飞猛进，人民生活显著改善，城乡面貌发生了翻天覆地的变化。面对改革开放取得的伟大成就，我们倍感骄傲和自豪，更加坚定了对中国特色社会主义的道路自信、理论自信、制度自信、文化自信，更加坚定了新时代继续推进改革开放、为实现中华民族伟大复兴中国梦不懈奋斗的决心和信心。

一年来，在省委、省政府和市委的正确领导下，市政府以习近平新时代中国特色社会主义思想为指导，深入贯彻党的十九大精神和习近平总书记视察甘肃重要讲话及“八个着力”重要指示精神，坚持稳中求进工作总基调，以供给侧结构性改革为主线，突出抓重点、补短板、强弱项，全市经济平稳增长、民生持续改善、社会安定和谐，朝着全面建成小康社会的目标迈出了新的步伐。预计全市完成生产总值690亿元，增长3%；社会消费品零售总额254亿元，增长8%；财政总收入157.7亿元，增长5.7%；一般公共预算收入53.1亿元，增长13.5%；城镇居民人均可支配收入29674元，增长8%；农村居民人均可支配收入8887元，增长9.5%。

——精准扶贫取得明显成效。新建贫困村专业合作社695个，实现了贫困村全覆盖。“331＋”产业扶贫模式带动贫困户3.67万户，当年分红6524万元。175个贫困村退出，8.7万人脱贫，贫困发生率由上年的9.3%下降到5.5%。西峰区、正宁县正式摘掉了贫困县帽子。

——生态产业发展势头良好。引进建办龙头企业30户，草畜、苹果、中药材产业化水平明显提升。游客人数和旅游收入分别增长29.5%和30%。开采天然气1.07亿立方米。并网风电65万千瓦，光电8万千瓦。在第三方平台注册活跃电商网店1150家，线上销售增长40%。

——招商引资取得较大突破。全年新签约项目79个，落地开工67个，开工率84.8%，创近年新高。天津天士力、广州立达尔、陕西盘龙、福建圣农、正大集团、大连万达、今日阳光、深圳五方等一批知名企业先后落户我市。

——重大项目建设顺利推进。实施500万元以上项目562个，其中5000万元以上项目132个。银西高铁、甜永高速、打庆高速、海绵城市、扬黄工程、华为云计算大数据中心等事关庆阳长远发展的重大项目正在顺利实施。

——民生保障更加普惠具体。省列10件、市列8件民生实事办结完成。城镇新增就业3.9万人，城镇登记失业率2.7%。落实各类教育资助资金8.58亿元，惠及40多万名学生。全民基本医保参保率达到98%，养老保险续保率达到97.3%。公立医疗机构全面实行“先诊疗、后付费”和“一站式”结报，贫困群众合规医疗费用报销比例达到85%。为40.58万名困难群众发放低保、医疗救助等各类救助资金8.07亿元。

——“放管服”改革步伐加快。取消调整行政审批事项49项，取消中介服务事项114项，清理取消各类证明事项140项。“多证合一”全面落实，“四办”改革力度加大，市级政务服务事项96.8%进驻政务大厅、96.1%可网上办理、50%以上可一窗办理，企业开办时间压缩至5个工作日以内，政府投资工程建设项目审批时间压减一半。

一年来，我们突出抓了七个方面的工作：

一是全力推进脱贫攻坚。全年一般公共预算扶贫支出29.4亿元，增长97%。投放特色产业发展工程贷款13.74亿元。坚持“四类分类”，为2.81万户一、二类贫困户落实奖补资金5.93亿元，为3.01万户三、四类贫困户落实奖补资金和贴息贷款6.88亿元，使“一户一策”帮扶措施更精准、落实更有效。全面推广“331+”产业扶贫模式，贫困户入股合作社、企业股金8.45亿元。实施农业保险14类19种，覆盖贫困户所有种养产业。全省产业扶贫现场推进会在我市召开。培训贫困劳动力3.3万人，输转4.09万人，开发农村公益性岗位5979个。建办扶贫车间23个。开工建设农村公路1455公里，实施各类饮水工程9177处，治理供电瓶颈自然村155个，光纤网络实现行政村全覆盖。建设易地扶贫搬迁安置住宅4226套，实施贫困户危房改造9409户。建成省市级美丽示范村51个、环境整洁村120个。省市县各级帮扶单位深入开展“六查三问一细化”活动，协调落实帮扶资金2.1亿元。天津市河东、南开、红桥、北辰、静海5区及中央党史和文献研究院、国家开发投资集团公司、中国化学工程集团公司，投入帮扶资金2.11亿元。91户民营企业和商会结对帮扶115个贫困村组，投入资金1.2亿元。

二是培育壮大产业基础。农业产业发展势头强劲。新建万只种羊繁育场6个、千只种羊扩繁场100个、千只规模育肥场212个，肉羊年屠宰加工能力达到130万只。3600万羽肉鸡生产线启动运营。15万头生猪示范项目建成投产。海越苹果分拣线建成投运，新建果蔬保鲜库71座、储藏能力2.2万吨，“庆阳苹果”获国家级农产品地理标志创建示范样板，郑州商品交易所在我市设立了苹果期货交割仓库。特色农产品进入北京家乐福、武汉中商、上海果品公司等16家商场超市。种植中药材27万亩，青贮饲草312万吨。粮食总产量达到141.6万吨，增长10.5%。能源化工产业取得新进展。长庆油田建设步伐加快，华北油田镇原区块复产，玉门油田环县木西区块启动产能建设，原油产量完成816万吨，加工量完成312万吨。庆阳瑞华天然气综合利用及调峰项目、永欣石化石油树脂项目建成试运行。甜水堡2号矿井启动联合试运转，核桃峪矿井三期、新庄矿井二期工程持续推进。文化旅游产业快速发展。参加央视《魅力中国城》栏目宣传竞演，荣获“2018年度十佳魅力城市”称号，庆阳香包被评为“2018年度魅力旅游文创产品”。药王洞养生小镇晋升为4A级景区，山城堡战役纪念园和黑老锅景区晋升为3A级景区，17个村列入全省乡村旅游示范村三年建设目录。

三是不断强化基础支撑。扬黄工程向庆城供水项目建成运行，五台山、莲花寺水库完成主体工程，小盘河水库混凝土坝段封顶。马莲河水利枢纽工程可研报告通过水利部审查。庆中、洪德330千伏输变电工程加快建设。实施和谐广场改造等海绵城市项目49个，新区雨洪集蓄工程、城南生态绿地公园建成投用。庆化大道、北京大道延伸段建成通车，新建停车场5处，市区3座人行天桥即将建成。东区污水处理厂、污泥处理厂建设持续推进。实施城镇棚户区改造5313户，基本建成5283户。

四是持续改善生态环境。子午岭自然保护区、县级以上饮用水源保护区油井全部封堵退出。建成垃圾填埋场19个、垃圾转运站1532座、污水处理站52座，河流沿线污水直排、垃圾倾倒和河道采砂问题得到进一步治理，妥善应对处置2起河流污染事件。剔除沙尘天气影响后，市区优良天气率达到95.8%。深入推进“再造一个子午岭”工程，造林100.3万亩。经过多年不懈争取，黄土高原沟壑区“固沟保塬”综合治理规划获得水利部正式批复，当年新修梯田23.2万亩。

五是统筹发展社会事业。创建省级创新型试点城市、国家知识产权强市试点城市、国家农业科技园区获得批准。实施1191个“改薄”单体项目，建成81所学校“温暖工程”。普通高考二本以上进线率35.1%，提高2个百分点。市妇女儿童医院完成主体工程，庆阳大剧院建成投用，庆阳传媒中心即将建成。加强全科医生培训，引进紧缺专业人员141人。城乡低保特困人员救助供养标准和退休人员基本养老金标准得到提高，机关事业单位基本养老保险制度改革稳步推进。

六是深入推进改革开放。“放管服”改革持续深化，建立了“12345”市长热线。顺利实现市县两级“三保”机构整合和城乡居民“两保”制度整合。公立医院改革不断深化，再次被国务院通报表扬。加快农村“三变”改革，农村土地确权颁证基本完成，盘活集体资产13亿元，累计流转土地166万亩。“331+”产业扶贫模式入选中国经济体制改革杂志社“改革开放40年地方创新40案例”。镇原、环县城乡建设用地增减挂钩项目首批交易3651亩。商事制度改革深入推进，新增市场主体1.33万户。减费降税措施有效落实，取消行政事业收费30项、停征26项，减少企业增值税9615万元，一般工商业用电价格平均降低10%。对外开放合作进一步扩大，与加拿大沙威尼根市缔结为友好城市，荣获“国际友好城市交流合作奖”。庆阳机场新增4条航线，全年旅客吞吐量达到42万人次。

七是创新加强社会治理。深入推进文明城市创建，提升城市管理水平，综合治理高价彩礼等陋习，推动移风易俗。市级应急指挥平台建成投用，“雪亮工程”全面建成，“扫黑除恶”专项斗争取得阶段性成效，社会治安持续好转，安全生产形势稳定向好。民族乡村加快发展，宗教领域突出问题得到有效整治。法治宣传教育持续推进，“巧儿说法”法治品牌获得国家商标认证。

过去的一年，我们落实全面从严治党要求，加强政府系统党风廉政建设，深入开展“转变作风改善发展环境建设年”活动，持之以恒纠正“四风”，“三公”经费下降3.8%。严格执行政府工作规则和重大事项决策程序，主动向市委常委会汇报工作，自觉接受人大依法监督、政协民主监督和社会各界监督，办理人大代表建议109件、政协委员提案283件。进一步强化审计监督和政府督查，从严从实整改国务院大督查、省委巡视、中省环保督察、扶贫领域核查、安全生产检查和各级审计发现反馈的问题，各项工作不断改进。

各位代表，去年在经济形势稳中有变、变中有忧的情况下，我市改革发展成就实属来之不易。这是省委、省政府和市委正确领导的结果，是市人大、市政协和社会各界鼎力支持的结果，是全市人民同心同德、真抓实干的结果。在此，我谨代表市人民政府，向各位代表和政协委员，向全市各行各业的劳动者，向各民主党派、工商联、无党派人士和人民团体，向驻庆部队和中央、省属驻庆单位，向在庆投资兴业的外地客商，向所有关心支持庆阳发展的朋友们，表示崇高的敬意和衷心的感谢！

在总结成绩的同时，我们也清醒地认识到，工作中还存在诸多困难和问题。主要是：实体经济困难较多。煤电项目建设进展缓慢，庆化公司600万吨扩能改造等能源项目推进受阻。庆化公司周期性停产检修，致使生产总值、工业增加值和财政收入受到较大影响。中小企业运营困难、效益不高，一些甚至处于停产状态，经济活力减弱，预计全市规模以上工业增加值完成307亿元，下降2%。项目投资持续下滑。金融风险管控趋紧，政府举债受限，企业融资困难，民间投资意愿不高，一些项目建设进展缓慢，南太高速、董志塬水厂等一批项目未能按计划实施。预计地方固定资产投资完成218亿元，下降22%。公共服务保障不足。市县（区）政府债务较重，保基本保运转压力增大，民生领域投资不足。市区择校热、大班额、上学难问题尚未有效缓解，供热、供气、供水等公共保障还不到位，特别是供热问题长期积累，成为市民反映强烈的热点。扶贫领域问题不容忽视。农民专业合作社的带动能力不强，贫困群众内生动力不足，脱贫增收的产业基础还不稳固，群众身边的“微腐败”时有发生。收入水平略高于贫困线的群体缺乏政策支持。部分扶贫贷款未用于产业发展，如期还贷存在风险。工作落实还有不小差距。形式主义、官僚主义依然存在，文件多、会议多、检查考核多，耗费了大量时间和精力。一些干部思想有顾虑，不敢大胆工作，不愿担当负责，甚至失职渎职，导致一些工作落实不到位，一些问题整改不彻底，一些应该办好的事情没有办好。对这些问题，我们必须正视，主动担当，深入研究，采取措施，认真加以解决。

2019年目标任务

今年是新中国成立70周年，是全面建成小康社会的关键之年。从大势看，我国发展仍处于并将长期处于重要战略机遇期，国家推进科技创新、制造业高质量发展、新旧动能转换、绿色发展、改革开放，将为我市发展赋予新的动能。从政策看，国家将加大减费降税力度，较大幅度增加地方政府专项债券规模，加大基础设施领域补短板力度并向中西部地区倾斜，将为我市发展带来新的机遇。从市情看，石油、煤炭、天然气等能源产业开发力度将持续加大，大数据及其相关战略新兴产业正在培育，现代农业、文化旅游加快发展，交通、水利等基础设施不断改善，必将为我市经济社会发展提供有力支撑。按照中央、省委省政府和市委的决策部署，统筹考虑宏观经济形势和我市发展实际，2019年市政府工作的总体要求是：坚持以习近平新时代中国特色社会主义思想为指导，全面贯彻落实党的十九大和十九届二中、三中全会，中央经济工作会议，习近平总书记视察甘肃重要讲话和“八个着力”重要指示精神，统筹推进“五位一体”总体布局，协调推进“四个全面”战略布局，坚持稳中求进工作总基调，坚持新发展理念，坚持推动高质量发展，坚持以供给侧结构性改革为主线，坚持深化市场化改革、扩大高水平开放，抓实“六稳”举措（稳就业、稳金融、稳外贸、稳外资、稳投资、稳预期），落实“八字方针”（巩固、增强、提升、畅通），继续打好“三大攻坚战”，全力推进以石油煤炭天然气为主的能源产业发展，加快实施乡村振兴战略，持续加强基础设施建设，全面深化改革开放，不断提高民生保障水平，全力维护社会和谐稳定，为全面建成小康社会打下决定性基础，以优异成绩庆祝新中国成立70周年。

经济社会发展预期目标是：在完成各项约束性指标的基础上，生产总值增长4%，地方固定资产投资总额增长6%，社会消费品零售总额增长7%，财政总收入增长6%，一般公共预算收入增长5%，城镇居民人均可支配收入增长6.5%，农村居民人均可支配收入增长9%。

重点做好八个方面的工作:

（一）强力攻坚精准脱贫，夯实乡村振兴基础

坚持把脱贫攻坚作为压倒一切的政治任务和实施乡村振兴战略的“头号工程”，盯紧深度贫困县乡，盯紧特殊困难群众，坚持分类指导，抓实“一户一策”，提高脱贫质量，确保实现庆城、华池、合水、宁县4个县摘帽，285个贫困村退出，10.29万人脱贫。

进一步做实产业扶贫。健全完善生产组织、资金投入、产销对接、风险保障体系，着力提升产业扶贫效益。大力培育发展龙头企业，确保每个特色优势产业都有1个以上带贫能力强的龙头企业，实现龙头企业对特色产业合作社全覆盖。规范提升专业合作社，推行产业指导员制度，加强合作社理事长培训，完善民主管理机制和财务制度，鼓励专业大户和有本事的人加入、领办合作社，确保所有贫困村合作社运营规范、增收增效。全面推广“331+”产业扶贫模式，注重发挥市场作用，建立企业、合作社、贫困户之间稳定的产销合作关系和利益联结机制，持续做强草畜、苹果、瓜菜、苗林等主导产业，着力发展小杂粮、中药材等特色产业，进一步扩大产业规模，提升质量效益。加强农产品冷链物流设施建设，新建果蔬保鲜库29座，投放移动保鲜车27辆。

提升就业扶贫质量。实施就业扶贫三年行动计划，统筹整合各类培训资源，切实提升贫困群众就业技能，提高劳务输转的组织化程度。鼓励兴办多种形式的扶贫车间，建设乡村供销加油点，积极开发乡村公益性岗位，帮助贫困劳动力在家门口就业增收。

补齐公共服务短板。强化义务教育控辍保学力度，落实教育扶贫特惠政策，确保贫困家庭适龄学生不失学辍学。落实健康扶贫特惠政策，细化“一人一策、一病一方”，实施家庭医生签约服务和送医上门，不断提升贫困群众健康保障水平。改造建档立卡贫困户、低保户、分散供养特困户、贫困残疾人家庭等重点对象危房3554户，基本实现农村安全住房全覆盖。新建集中供水工程33处、分散供水工程2200处，全面解决农村群众饮水安全问题。新建农村公路355公里，实施公路安全防护工程639公里。实施农网升级改造项目，解决深度贫困村“低电压、卡脖子”问题。建设高标准农田110万亩。

持续改善农村人居环境。坚持美好环境与幸福生活共同缔造，实施农村人居环境整治行动，新建一批污水、垃圾处理设施，确保垃圾定点收集、按时清运、规范处理。扎实开展村庄绿化美化，推进“厕所革命”，加快废旧宅基地复垦整理，推动村庄公共照明设施建设，加快创建美丽示范村、环境整洁村，建设生态宜居村庄。

大力培育文明乡风。坚持扶钱扶物与扶志扶智有机统一，充分发挥农村培训阵地作用，讲好习近平总书记扶贫重要论述，讲好惠农政策，讲好农业实用技术，全面提升农民综合素质，增强脱贫造血功能。发挥村民议事会等群众组织作用，加强对高价彩礼、薄养厚葬、赌博迷信等陈规陋习的专项治理，切实推进移风易俗。发布道德“红黑榜”，完善扶贫领域诚信监管体系，将不履行赡养义务、虚报冒领扶贫资金、严重违反公序良俗等行为人列入失信人员名单，教育引导群众增强尚德守信意识。

用心做好帮扶工作。全面落实脱贫攻坚帮扶工作责任，充分发挥驻村工作队作用，扎实开展“六查三讲一强化”和“十问十推进”活动。积极对接天津市对口帮扶和中省单位定点帮扶，实施好东西扶贫协作三年行动计划，深入推进“百企帮百村”行动，落实好产业合作、劳务协作、人才培养等项目。坚持脱贫不脱帮扶、脱贫不脱政策、脱贫不脱项目，巩固和扩大脱贫成果。

（二）发展绿色生态产业，推动实体经济提质增效

坚持生态优先、绿色发展，大力推动传统产业转型升级，重点发展特色生态产业，促进质量变革、效率变革、动力变革。

集群化发展现代农业。支持中盛农牧、正大集团、福建圣农等现代畜牧业龙头企业，加快推进肉羊、生猪、肉鸡产业化项目建设，力争饲养量均增长20%以上。积极筹办好第十六届中国羊业发展大会，促进羊产业快速健康发展。支持荟荣草业等企业大力发展饲草产业，加大秸秆青贮力度，更新种植紫花苜蓿100万亩。支持天津天士力、陕西盘龙等企业发展中药材标准化种植，建设万亩示范基地2个、5000亩示范基地4个。持续推广“海升”模式和“西农”技术，推进无毒苹果苗木繁育和苹果标准化生产、品牌化销售，新栽苹果10万亩。支持中庆公司带动种植籽用南瓜20万亩。种植高原夏菜和设施瓜菜110万亩。加快注册农产品“三品一标”，打响庆阳苹果、湖羊、道地中药材、小杂粮等区域公用品牌。落实耕地保护制度，推行轮耕轮休，防止耕地撂荒，保障粮食安全，粮食总产量稳定在140万吨左右。积极开辟销售渠道，帮助农户消化过剩苗木。加大牧草机具购置补贴力度，提升农业机械化和农机装备水平。

绿色化开发传统能源产业。推动创建绿色园区、绿色工厂，开发绿色产品，提高能源利用效率。支持长庆油田、华北油田、玉门油田扩能上产，力争原油产量达到840万吨以上。支持庆化公司扩能改造项目。力争庆化公司原油加工量达到360万吨以上。支持刘园子煤矿加快复产，甜水堡2号矿井正式投产，核桃峪矿井完成三期工程、力争具备联合试运转条件，新庄矿井完成二期工程，全年新增原煤产量80万吨。确保陇东至山东±800千伏特高压直流外送工程获得核准，力争开工建设，破解制约煤电产业发展瓶颈。

加快发展清洁能源产业。推进“气化庆阳”气源工程主干管道项目，建成庆城至长庆桥输气管线，开工建设环县至庆城、长庆桥至宁县输气管线，全年生产天然气、石油伴生气3亿立方米，加工压缩天然气2000万立方米、液化天然气3.2亿立方米，天然气储气能力达到1200万立方米。建成西峰垃圾焚烧发电项目一期工程并投产运行。紧跟特高压直流外送通道建设，大力开发风能光能资源，大力发展风电、光伏产业。

打造全域文化旅游产业。突出大景区建设，推动南梁创建5A级景区、北石窟驿创建4A级景区，加快蒲河川、湘乐川、茹河川3条景观廊道建设，高效推进马岭黄酒古镇、环州古城、黄帝文化博览园等重点项目，完善景区基础设施和服务功能。因地制宜发展乡村旅游，依托农耕文化、田园风光、村落民宅、生态农业等特色优势，新建乡村旅游示范村6个、农家乐42个，打造肖金幸福小镇和驿马、山庄湖羊小镇等生态田园综合体。盘活推进民俗文化产业园建设，发展香包刺绣、皮影、剪纸等民俗文化产业。力争文化产业增加值增长10%，旅游综合收入增长25%。

培育新兴产业。实施好电商进农村综合示范项目，支持推进“快递下乡”，有效组织农产品网络销售，力争全年电商交易额增长20%。加快肖金物流园建设。建成华为云计算大数据中心，培育发展大数据产业。鼓励医养结合、文化娱乐、休闲健身等新兴消费，发挥消费对经济增长的拉动作用。

推动科技创新。加快国家农业科技园区建设，启动科技创新孵化中心、海南良种繁育基地建设，推动国家知识产权强市试点城市、省级创新型城市创建，加强院地、校地合作，强化应用研究，鼓励企业增加研发投入，激励科技人员开展技术研发和先进实用技术推广，努力促进科技创新成果向现实生产力转化。

（三）切实优化营商环境，毫不动摇支持非公经济发展

牢固树立“产业第一、企业为大”的理念，把民营企业家当作“自己人”，真情实意搞好服务，真金白银支持发展。

强化政策扶持。梳理公布扶持民营企业发展的优惠政策清单，全面落实市场准入负面清单制度，确保民营企业与国有企业在市场准入、融资、用地等方面享受同等待遇，平等获得各种资源。全面落实减费降税政策，降低电价和物流成本，实行工业用地弹性出让制度，有效减轻企业负担。加大财政扶持力度，持续落实小微企业创业促就业财政补贴政策，落实企业商标、品牌、质量和专利方面的奖励政策。补贴扶持1000名高校毕业生到民营企业就业，对民营企业引进的人才，平等享受政府有关待遇保障。大力弘扬民营企业家自律自强精神，使扶持政策发挥最大作用。

加强权益保护。全面推行“双随机、一公开”监管，规范执法行为，减少对企业的各类检查，确保民营企业合法财产不受侵犯、正常经营不受干扰。建立市场化的债权债务盘活机制，依法依规全面清理政府拖欠企业债务问题，积极化解企业“三角债”。深入开展民营企业帮扶专项行动，落实领导干部联系帮包企业制度，定期召开民营企业发展联席会议，及时掌握企业诉求，回应企业关切，落实“一企一策”帮扶措施，切实为企业排忧解难。

破解融资难题。加强“政银担企”合作，引导金融机构加大对非公经济支持力度，推动小微企业贷款扩量降本。充分发挥金控融资担保公司枢纽作用，用好并逐步增加县（区）政府风险补偿金，为企业提供市场化、多层级的融资服务，提高特色产业发展工程贷款利用率。利用中小企业直接融资增信基金和“助保贷”“互助贷”政策，撬动金融资金和社会投资。支持企业通过上市、“新三板”挂牌、产权转让、发行债券等方式直接融资，拓宽融资渠道。

推动转型升级。围绕农特产品精深加工、建筑建材、中药制品、机械制造等重点产业，培育和发展一批地方骨干企业。支持西峰制药公司搬迁改造，支持百跃古象、陇牛乳业、醋头醋、京酿食品、长荣机械等成长性较好的企业，向专业化、精细化、特色化方向发展，争创行业“小巨人”。扶持引导小微企业扩大生产经营规模，逐步成长为骨干企业。鼓励企业完善法人治理结构，提升核心竞争力。加快园区“七通一平”基础设施建设，搭建人才、科研等优势资源和生产要素平台，吸引更多企业入驻园区、投资兴业。

（四）持续扩大项目投资，着力改善基础设施条件

认真研究国家拉动有效投资、加快补短板的政策导向，分门别类谋划储备、争取实施一批重大项目，努力提升基础设施供给质量。

加强城市建设和管理。以创建文明城市、海绵城市、生态城市、智慧城市为抓手，统筹发展中心城区、县城和小城镇，高标准规划，科学化建设，精细化管理，全力推动城市出形象、管理上水平、文明迈大步。加快实施海绵广场、海绵道路、海绵学校和内涝治理等项目。建成城东污水处理厂、污泥处理厂，完成南部工业园区污水处理再生利用主体工程。新建大型停车场5处，引导住宅小区地下停车场以租代售，缓解停车难问题。加快推进棚户区改造，加强背街巷道、居民小区出入口治理。提升供水保障能力，逐步解决水质安全和二次供水问题。切实加强供暖保障，提前维护设施、敷设管道，绝不能再出现大面积供不上热、温度不达标问题。全面推行垃圾分类制度，规范生活垃圾收集转运处置。推进城市沿街楼宇、公共场馆、广场游园等重点区域亮化、美化。深入开展户外广告整治，切实加强户外广告管理。及时维护人行道、路灯、标识牌等设施，优化公交线路，整治交通秩序，不断提升城市管理水平和文明形象。

加快建设立体交通网络。全力配合银西高铁庆阳段建设，高标准推进站前广场和配套枢纽工程。力争建成华池通用机场。加快甜永、打庆高速公路，环县二十里沟口至车路崾岘一级公路，孙家湾至白马、阳路湾至席沟圈、板桥至杨坪三级公路建设。争取南太高速公路开工建设。完成庆阳至平凉高速公路前期工作，争取早日开工。积极推进庆阳至平凉铁路、褚家湾运煤通道前期工作。

推进重大水利电力工程建设。完成小盘河水库主体工程，建成五台山、莲花寺2个水库并投入运行。下决心建设扬黄工程向市区延伸供水项目，从根本上解决市区供水安全问题。力争马莲河水利枢纽工程通过国家发改委审查，完成莲花寺水库引水工程前期工作。建成庆中、洪德330千伏输变电工程，为银西高铁提供电力保障。

提高通讯服务水平。大力推进光网建设，优化公路隧道、地下空间、电梯、生产经营活动场所、林区等重点区域4G网络深度覆盖，提升电信基础设施整体水平。

（五）加强环境治理，推进生态文明建设

坚持节约优先、保护优先，落实最严格的环境保护制度，努力建设山清水秀、天蓝地绿、空气清新、环境整洁的美丽庆阳。

积极推进国土绿化行动。深入实施“再造一个子午岭”工程和天然林保护、退耕还林、三北防护林、森林抚育等重点生态项目，造林105万亩。落实黄土高原沟壑区“固沟保塬”综合治理规划，推进北部山区多沙粗沙区、巴家嘴库区上游等重点区域综合治理，新修梯田10万亩，修建小型水保拦蓄工程300座（处）。严格实行封山禁牧，促进生态自然修复。

持续加强污染防治。有序推进城乡冬季清洁供暖，逐步对城乡结合部及周边乡镇居民土炕、土灶、小火炉实施煤改气、煤改电、洁净煤替代工程。建立秸秆收储体系，推动秸秆利用饲料化、肥料化。严格监控建筑工地扬尘污染，大力发展绿色交通，积极推广清洁能源，基本消除重污染天气。严格落实“河长制”“湖长制”，突出泾河、马莲河等重点河流污染治理和城乡黑臭水体治理，完成县级以上地表水水源地清理整治工作，强化餐厨废弃物处置，大力推动畜禽养殖废弃物资源化利用，提升水环境质量。扎实开展土壤污染调查摸底，持续抓好庆化公司老厂区、原马岭炼油厂等油田废弃场地治理修复工作，有效防范土壤污染风险。

健全生态保护机制。坚持“谁受益、谁补偿”原则，建立生态环境保护补偿治理机制，探索建立跨区域污染补偿办法。完善环境监察体系，加大重点区域、流域、敏感节点和重污染行业的环境执法检查力度。健全举报、听证、舆论和公众监督等制度，提高公众环保意识和行动自觉。积极倡导绿色生活方式和消费模式，推进绿色居住、绿色出行，推广使用绿色节能产品。反对铺张浪费、过度包装和奢华消费，实现生活方式和消费模式向节约适度、绿色低碳转变。

（六）深化改革开放，增强经济社会发展活力

紧盯重点领域，抓住关键环节，破解发展难题，以改革增活力，以开放促发展。

推进政府机构改革。认真做好涉改单位人员转隶工作，教育和引导干部职工正确对待进退留转，确保改革期间各项工作不断档、不缺位。认真做好资产清理登记和处置工作，防止国有资产流失。合理调整办公用房，保证人员及时到位，确保按期完成机构改革各项任务。

推进“放管服”改革。持续转变职能、简政放权，再取消和下放一批行政审批事项。坚持管人与管事相结合，将县（区）能管好的事项尽量下放，靠实责任主体，防止监管缺位。进一步理顺市、区权责关系，推动人权、事权、财权相统一，建立科学高效的城市管理体制。深入推进“四办”改革，进一步压缩办事流程，创新服务方式，切实为企业松绑、为群众解绊。推动各级政务大厅服务升级，实现政务服务事项应进必进。加快建设政务服务一体化综合应用平台，实现数据资源跨部门共享应用，让数据多跑路、群众少跑腿。推行政府投资项目网上申报、并联审批、信息公开、协同监管，提高项目审批效率和服务质量。持续抓好商事制度改革，实施“证照分离”，进一步减证便民。稳步推进农业、交通、市场监管、城市管理等领域行政综合执法改革，提高监管效能。

推进农村综合改革。持续深化“三变”改革，完善农村土地产权交易市场，推动城乡建设用地增减挂钩。巩固农村产业发展资金专业合作社试点成果，探索开展“多县一行”制村镇银行试点，推动农村金融综合服务室建设，继续推进农业保险“增品扩面提标降费”试点。加快集体经营性资产股份合作制改革，不断壮大村级集体经济。

推进财税体制改革。强化预算约束，加强绩效管理，不断提高财政资金使用效益。推进教育、医疗、就业等基本公共服务领域省以下财政事权和支出责任划分改革，完善市县（区）政府基本公共服务财力保障体系。

推进其他领域改革。理顺全市工业集中区管理运行体制。强化国有资产统一监管体系，推进国有企业脱钩改制，加快建立现代企业制度。深化公共资源交易改革，加快打造统一高效的交易平台体系。深化医药卫生体制改革，全力落实分级诊疗、现代医院管理、全民基本医保、药品供应保障、综合监管制度。推进户籍制度改革，加快农业转移人口市民化进程，城镇化率提高1.5个百分点。

加大招商引资力度。坚持把招商引资作为培育新动能的第一抓手，积极走出去、请进来，持续在招大引强上求突破。市县（区）政府、相关部门和园区主要负责人要腾出一定时间精力，亲自带队外出推介招商。加强同“一带一路”沿线国家、城市的合作交流，主动融入关中平原城市群，积极参加各类重要节会，大力推动庆阳产品走向全国、全球大市场。坚持精准招商、产业链招商、以商招商、委托招商，打有把握之仗，招有实效之商。完善招商引资优惠政策，建立招商奖励制度，坚决兑现招商承诺。实行一站式办公、保姆式服务，让企业提清单，由政府做答案，帮助解决项目落地建设环节的实际困难。

（七）积极回应群众关切，不断增进民生福祉

坚持以人民为中心的发展思想，针对群众最急最忧最盼的问题,尽力而为，抓好保障和改善民生各项工作。

优先发展教育事业。支持集团化、一体化办学，建立学校联盟，促进教育资源均衡。深入实施学前教育行动计划，加快普及高中阶段教育，推动发展现代职业教育，持续提升特殊教育，支持陇东学院加快应用型大学建设，尽力帮助解决学院发展中的困难和问题。统筹乡村小规模学校和乡镇寄宿制学校建设，实施农村中小学“温暖工程”，改善办学条件。优化市区教育布局，实施市实验小学整体搬迁、团结小学新校区建设等项目，逐步缓解择校热、大班额、上学难等问题。重视教师培养培训，加强师德师风建设，打造高素质教师队伍。

繁荣发展文化艺术事业。传承优秀传统文化，推进文化遗产“历史再现”工程，申报文化生态保护区项目。新建8个特色博物馆。建成庆阳传媒中心并投入使用，继续推进市图书馆、文化馆和画院项目前期工作。加大政府购买公共文化服务力度，编创一批文艺精品力作，开展“千台大戏送农村”“文化下乡”“文艺五进”等文化惠民活动，不断丰富群众文化生活。

推进健康庆阳建设。加强紧缺医疗人才培养和引进，优化医疗卫生服务，综合防治重点传染病、慢性病和地方病，推进基本公共卫生服务均等化。加快建设智慧医疗，推动医疗服务信息化。建成市妇女儿童医院，加快推进残疾人康复就业中心建设，谋划推进市精神康复医院前期工作。广泛开展全民健身运动，提升竞技体育水平。

加强就业和社会保障工作。强化职业技能培训，鼓励创业带动就业，城镇登记失业率控制在4%以内。实施全民参保计划，推进基本医疗和基本养老保险法定参保对象全覆盖，提高工伤、失业、生育保险参保率。聚焦城乡特困人群，做好社会救助工作。落实城镇困难职工解困脱困三年行动计划，逐步解决5115户建档立卡职工生活困难。大力发展社会福利和慈善事业，提升养老服务质量。加大农村留守人员的社会保护与关爱，完善困难残疾人生活补贴和重度残疾人护理补贴制度，着力维护弱势群体合法权益。

精心办好8件民生实事。(1)开展农村人居环境整治，推动全域无垃圾专项治理，建设一批美丽示范村、环境整洁村和“厕所革命”示范村。(2)对西峰城区部分老旧巷道及老旧小区供水管网进行提质改造。(3)建设市级全民健康信息平台，接通省级平台，实现市县公立医院互联互通。(4)在二级以上医院建设“急救绿色通道”，在人群密集公共场所配备自动体外除颤器。(5)对全市35-64周岁的农村妇女免费进行“两癌”检查。(6)升级改造市区重要路口及人行道交通信号灯。(7)为全市70周岁以上老年人每人投保意外伤害保险20元。(8)对参加当年高考并被二本及以上院校录取的城乡困难家庭大学生进行一次性资助。

（八）加强各类风险管控，维护社会和谐稳定

坚持底线思维，强化风险意识，未雨绸缪，抓早抓小，防患于未然，确保不发生区域性、系统性重大风险。

提高社会治理水平。培育和践行社会主义核心价值观，规范市民行为，弘扬时代新风。深入开展法制宣传教育，提高全民法律素质。完善信息化、立体化社会治安和公共安全防控体系，纵深推进“扫黑除恶”专项斗争，严厉打击各类违法犯罪。做好信访维稳工作，坚持领导干部接访下访制度，畅通群众诉求表达渠道，依法有效化解矛盾纠纷和信访积案。做好退役军人工作，切实维护军人军属合法权益。做好民族工作，强化宗教管理，加强政策宣传，促进民族团结、宗教和顺。依法规范物业管理，维护业主合法权益。

强化安全监管。深化安全知识宣传教育，切实增强全民安全防范意识。全面落实安全生产党政领导责任、行业监管责任和企业主体责任，持续强化重点领域安全风险评估预警，排查整治隐患，坚决遏制较大及以上事故发生。加强应急预案演练，提升防灾减灾和应急处突能力。着力加强市场监督管理，强化食品药品、产品质量、特种设备安全监管，创建食品安全示范城市和农产品质量安全示范县。加强校园安全工作，深化校园周边环境综合治理，强化学校内部安全管理，积极开展学生安全教育和心理健康教育，有效防治校园欺凌，健全校园安全工作责任体系，切实保障师生安全和校园安定。

切实防范金融风险。加强金融领域潜在风险摸排和分析研判，加大不良贷款清收力度，严厉打击恶意逃废银行债务行为，规范金融市场秩序。清理整顿、规范发展小额贷款公司、融资担保公司、典当行等准金融机构，加强互联网金融监管，坚决打击取缔非法集资、非法金融活动。坚持“房子是用来住的、不是用来炒的”定位，逐步建立和完善房地产市场平稳健康发展的长效机制。严格落实政府债务管理规定，实行政府债务分级管理制度，坚守政府举债融资红线。杜绝政府隐性债务增量，稳妥化解存量。

加强政府自身建设

政贵在行，事成于实。新的一年，市政府将坚决贯彻全面从严治党部署，持续加强自身建设，努力建设人民满意的法治政府、创新政府、高效政府、廉洁政府和服务型政府。

一是提高政治站位。坚持以习近平新时代中国特色社会主义思想为指导，认真贯彻党的基本理论、基本路线、基本方略，树牢“四个意识”，坚定“四个自信”，坚决维护习近平总书记党中央的核心、全党的核心地位，坚决维护党中央权威和集中统一领导。严守党的政治纪律和政治规矩，自觉把政府工作置于党的领导之下，不折不扣贯彻落实党中央、省委和市委各项决策部署，全面正确履行政府职能，确保政令畅通、令行禁止。

二是严格依法行政。强化法治意识，带头维护宪法和法律权威，依法履行职责、行使权力。坚持重大决策公众参与、专家论证、风险评估、合法性审查、集体讨论决策制度，落实政府法律顾问制度，保障决策科学化、民主化。严格公正文明执法，推行行政执法公示、过程记录、重大执法决定法制审核制度，加强行政复议、行政应诉。自觉接受人大依法监督和政协民主监督，高质量办理人大代表建议和政协委员提案，主动听取各民主党派、人民团体和社会各界的意见建议。深化政务公开，全面接受社会监督，让权力在阳光下运行。

三是强化责任担当。坚持真抓实干、锐意进取，只要符合上级精神、庆阳实际和群众意愿，就大胆试、放手干。强化目标意识，全面推行政府工作项目化、责任清单化，让干部心中有“项目表”，肩上有“任务书”，手里有“施工图”。坚持严管和厚爱结合、激励和约束并重，对不作为、慢作为和弄虚作假行为，严肃问责追责，同时落实容错纠错机制，完善考核评价机制，激励政府工作人员放下包袱、积极作为，创造性开展工作。

四是提升行政效能。严格落实中央“八项规定”精神，严格落实省政府“十不准”要求，持续改进工作作风。大力精文减会，规范督查考核，纠正“烦琐哲学”，反对“事事留痕”，腾出更多的时间深入一线抓落实。严格落实首问负责、限时办结、公开承诺、及时反馈等制度，驰而不息治庸提能、治懒提效、治散提神、治慢提速，凡决定的事项必须落地，凡承诺的事情必须办成，切实提高政府的执行力和公信力。

五是坚持廉洁从政。严格落实全面从严治党主体责任，强化权力集中部门和高风险岗位廉政风险防控，规范政府采购、土地出让、工程招投标、政府投资等工作，不断扎紧制度的笼子。支持纪委监委依法监督执纪问责、加大违纪违法案件查处力度，推进审计监督全覆盖，营造风清气正的政治生态。

各位代表，新起点孕育新希望，新时代要有新作为。让我们更加紧密地团结在以习近平同志为核心的党中央周围，在省委、省政府和市委的坚强领导下，在市人大、市政协的监督支持下，同心同德、和衷共济，攻坚克难、砥砺前行，为加快建设幸福美好新庆阳而努力奋斗！
